# Application of Clustering Method to Explore the Correlation Between Dominant Flora and the Autism Spectrum Disorder Clinical Phenotype in Chinese Children

**DOI:** 10.3389/fnins.2021.760779

**Published:** 2021-11-24

**Authors:** Biyuan Chen, Na You, Bangquan Pan, Xueyi He, Xiaobing Zou

**Affiliations:** ^1^Child Development and Behavior Center, Third Affiliated Hospital of Sun Yat-sen University, Guangzhou, China; ^2^School of Mathematics, Sun Yat-sen University, Guangzhou, China

**Keywords:** autism spectrum disorder, gut microbiome, repetitive behavior, microbiome composition, alpha diversity

## Abstract

Autism spectrum disorder (ASD) is characterized by deficits in social interactions and repetitive, stereotypic behaviors. Evidence shows that bidirectional communication of the gut-brain axis plays an important role. Here, we recruited 62 patients with ASD in southern China, and performed a cross-sectional study to test the relationship between repeated behavior, gut microbiome composition, and alpha diversity. We divided all participants into two groups based on the clustering results of their microbial compositions and found *Veillonella* and *Ruminococcus* as the seed genera in each group. Repetitive behavior differed between clusters, and cluster 2 had milder repetitive symptoms than Cluster 1. Alpha diversity between clusters was significantly different, indicating that cluster 1 had lower alpha diversity and more severe repetitive, stereotypic behaviors. Repetitive behavior had a negative correlation with alpha diversity. We demonstrated that the difference in intestinal microbiome composition and altered alpha diversity can be associated with repetitive, stereotypic behavior in autism. The role of *Ruminococcus* and *Veillonella* in ASD is not yet understood.

## Introduction

Autism spectrum disorder (ASD) refers to early-onset neurodevelopmental disorders with core deficits in social interactions and restricted repetitive behavior and interest (RRBI) (Lord et al., 2018). Increasing evidence supports the notion that the gut-brain axis plays a non-negligible regulatory role in autism, while intestinal microbes are critical bidirectional communication components between the central nervous system (CNS) and our gut (Qin et al., 2010; Cryan and O’Mahony, 2011; Dinan and Cryan, 2013). Our brain can modify gastrointestinal dynamics and local blood flow, increasing or decreasing the secretion of gastrointestinal hormones, and thereby affecting the local immune response (Mayer, 2011; Dinan and Cryan, 2017). Simultaneously, external environmental signals are also mediated by the intestinal microbiome and interact in various ways with the CNS (De Vadder et al., 2014; Sherwin et al., 2018).

Recent studies have shown that the intestinal flora may directly affect our brain function by producing neuroendocrine metabolites such as short chain fatty acids (SCFAs) and tryptophan, or cause interference to other pathways that affect human physiological functions and behavioral performance, including stressful behavior, social interaction, eating, and addiction (Cryan and Dinan, 2012; Cussotto et al., 2018). Research has revealed that one of the gut microbes, *Lactobacillus rhamnosus*, diminishes anxiety-related behavior in rodent models depending on vagus signal transmission (Bravo et al., 2011). Social behavior deficits in germ-free (GF) mice are also considered critical evidence supporting the assertion that changes in the intestinal microbiome can affect brain function as well as behavioral changes in the individual (Desbonnet et al., 2014; Buffington et al., 2016).

Restricted repetitive behavior and interest (RRBI) is considered a key feature of ASDs and may be related to compositional changes of the intestinal flora (Wolff et al., 2014; McKinnon et al., 2019; Osadchiy et al., 2019), but few studies have addressed this domain (Ho et al., 2020). In this cross-sectional study, we hypothesized that the ASD gut microbiome would be reflected by similar community groups and the severity of their repeated, stereotypic behaviors would vary between clusters. Previous studies on intestinal flora alpha diversity in children with ASD have not yet reached a consensus on this (Liu et al., 2019). Thus, we hypothesized that alpha diversity may be related to one or more RRBI sub-domains.

## Materials and Methods

### Materials

#### Study Population

The research team recruited 62 subjects – (non-twin, from simple families, all skilled in Mandarin) – from southern China. All subjects were diagnosed with ASD according to the DSM-5 criteria by two physicians with extensive clinical experience, and their scores were further determined to be higher than the ASD cut-off by the Autism Diagnostic Interview-Revised (ADI-R) and Autism Diagnostic Observation Schedule (ADOS) assessment. Subjects age ranged from 3 to 10 years, with a male to female ratio of 3:1. The exclusion criteria for this study were genetic diseases with known definite chromosomal disorders, Rett syndrome, 22q13 deficiency syndrome, other degenerative symptoms, other serious neurological diseases (such as seizures, tuberous sclerosis) or severe mental illness, as well as children with significant congenital malformations or parents with serious illnesses. The study was approved by the Institutional Review Committee of the Third Affiliated Hospital of Sun Yat-sen University.

#### Microbiome Analysis

Parents used a clean disposable container or a non-contaminated urine-clean diaper to collect approximately 10 g of stool sample. Approximately 200 mg was placed in a sampling tube designed for fecal specimen collection (patent number: US 8202693, approved by Illumina for ensuring that the microbiota in human feces is stably stored for more than 1 week at room temperature). Parents returned the fecal specimens through a biological sample transport channel within 1 week and samples were stored at −80°C until analysis.

The 16S ribosomal RNA amplification sequencing of the V3–V4 gene region was performed on the Illumina Miseq X Ten high-throughput sequencing platform to identify and quantify bacterial taxa. Using Quantitative Insights Into Microbial Ecology (QIIME) software (Caporaso et al., 2010), bioinformatics analysis for the 16S ribosomal RNA amplification sequencing data was performed including using the closed-reference algorithm for operational taxonomic unit (OTU) classification unit screening, taxonomic assignment as well as alpha and beta diversity analysis.

**FIGURE 1:**
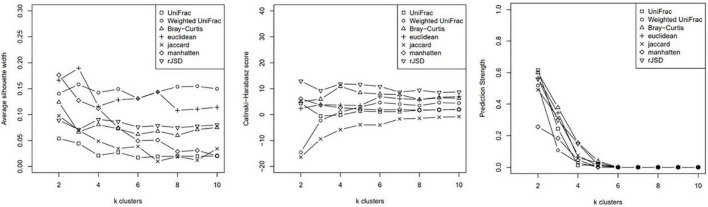
K-means of cluster index.

Clustering analysis was applied to the relative genus abundance of the 62 subjects using Partitioning Around Medoids (PAM) algorithm. The cluster scoring method was used to assess the optimal number of clusters (Figure 1).

#### Genera Analysis and Co-occurrence Networks

**FIGURE 2:**
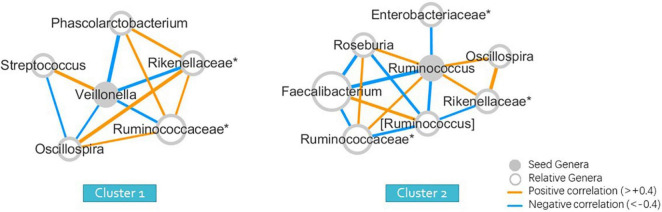
Seed-genera and co-occurrence networks between clusters (wunifrac distance). “*” means the unclassified genera, and specified in the family to which the genera belong.

The Wilcoxon signed-rank test was used to identify significant differences in the relative abundance of bacterial genera among clusters. The seeds of each cluster indicating the most significant representative genera were selected to form the co-occurrence networks using Spearman correlations (Figure 2) to elucidate the bacterial community dynamics.

### Autism Diagnostic Interview-Revised Assessment for Restricted Repetitive Behavior and Interest

The ADI-R is an appraisal-based, standardized, semi-finished, professional-use interview scale (Hus and Lord, 2013; Zander et al., 2017). It was used to differentiate “Autism Spectrum Disorder” in the DSM-5 diagnostic criteria and “Classic Autism” in DSM-4. A higher score indicates more severe symptoms in the field (Hus and Lord, 2013). A total of 14 behavioral items were included in the ADI-R assessment for classification and comparison of repeated stereotypies (Cuccaro et al., 2003; Mooney et al., 2009). This study used the ADI-R C-category scores to obtain the severity of social communication barriers and repetitive stereotypies in the form of parental interviews.

### Association of Within-Cluster Restricted Repetitive Behavior and Interest and Alpha Diversity

Alpha diversity is an indicator that describes the diversity of species within each individual (also known as local diversity). We used chao1, observed OTUs, Shannon and Simpson index to measure alpha-diversity for different clusters. Subsequently, Pearson correlation analysis was used to test all four measures of alpha-diversity for associations with repetitive, stereotypic behavior scores in the ADR-I assessment.

## Results

### Autism Spectrum Disorder Microbiota Were Clustered Into Two Groups

Based on the average silhouette width, Calinksi Harabasz score, and prediction strength, the subjects were clustered into two groups using the weighted-UniFrac distance and unweighted-UniFrac distance, which yielded better intergroup discrimination outcomes. Although *k* clusters of weighted- UniFrac indicated better between-group variance when *k* = 3 (Figure 1), the case number for each group was 19, 31, and 11. The power of phenotypic analysis was compromised because of the small sample size. After consulting statisticians, we performed subsequent analysis based on clustering into two groups using weighted- UniFrac (wunifrac) distances.

### Genera Analysis of the Two Groups of Seed Genera and the Construction of Co-occurrence Networks

To obtain a more intuitive comparison, we selected a representative seed genera and constructed a co-occurrence network for each cluster (Figure 2). The abundance of many genera was different from that of Clusters 1 and 2. The seed of each cluster refers to the one that differed the most between two clusters, with the lowest *p*-values of the Wilcoxon signed-rank test, with the maximum relative abundance in all samples greater than 0.001 and average relative abundance higher than the other group. The seed genus was *Veillonella* for Cluster 1, and *Ruminococcus* for Cluster 2, with *p*-values of 0.026 and 0.001 between two clusters, determined using the Wilcoxon signed-rank test, indicating statistically significant results (Supplementary Material 1). The members in the co-occurrence network were determined according to their Spearman correlations with the seed genera.

### Comparison of Characteristics Between Clusters

#### Demographic Characteristics

Our participants were recruited from a Han population in southern China. We used the weighted UniFrac distance (hereinafter referred to as wunifrac) to divide clusters for later comparisons. There was no significant difference between the two clusters upon baseline comparison of gender, age, breastfeeding, food allergies, frequency of probiotic or antibiotic intake, and preference for food (meat or vegetarian diets). See Supplementary Table 1.

#### Autism Repetitive Behavior Performance Difference Between Clusters

**FIGURE 3:**
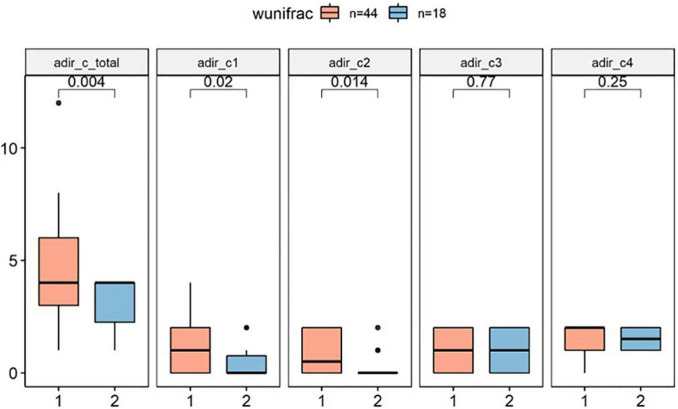
ADI-R C score difference between clusters (wunifrac distance). ADI-R, Autism Diagnostic Interview-Revised. ADI-R C score: Restricted, Repetitive, and Stereotyped Patterns of Behavior, adir_cl: encompassing preoccupation or circumscribed pattern of interest. adir_c2: apparently compulsive adherence to nonfunctional routines or rituals. adir_c3: stereotyped and repetitive motor mannerism. adir_c4: Preoccupation with parts of objects or non-functional elements of material.

In this study, we tested whether core phenotypes of autism differed between clusters by analyzing three independent domains of the ADI-R. In the RRBI (C score), our primary analysis indicated that Cluster 1 has significantly higher score than Cluster 2 (*p* < 0.01), implying that children in Cluster 1 had more severe repetitive stereotypic behaviors than the other group (Figure 3).

Subsequent secondary analysis, separately testing whether the RRBI sub-scale score entries of the repeated stereotypes sub-scale were different between clusters found that C1 scores (encompassing preoccupation or circumscribed patterns of interest) and C2 scores (apparently compulsive adherence to non-functional routines or rituals) were different between groups. However, there was no difference between clusters in the domain of social interaction (A score) and communication (B score) (*p* > 0.1; Supplementary Material 2).

#### Alpha Diversity Between Clusters

**FIGURE 4:**
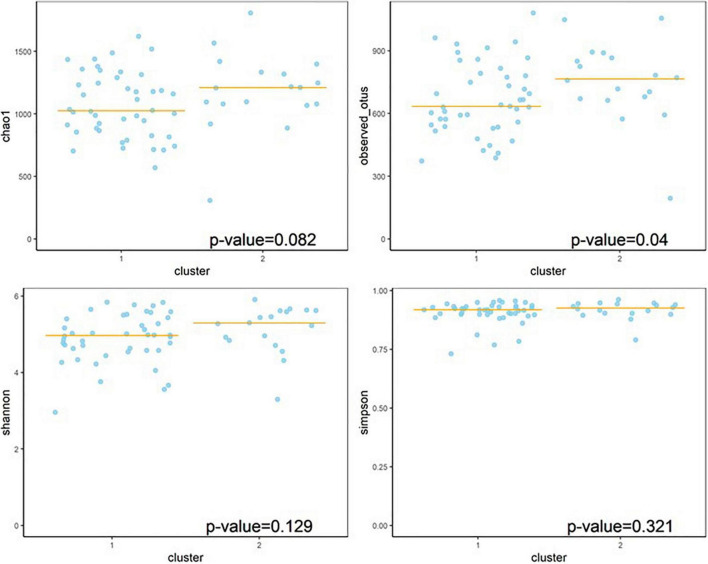
Alpha diversity between clusters (wunifrac distance).

The measurement of alpha diversity OS (observed OTUs) showed a difference (*p* = 0.04) between clusters, and Chao1 indicated a trend toward significance (*p* = 0.082), suggesting that Cluster 1 had less diversity and Cluster 2 had higher diversity (Figure 4). No between-cluster differences were found using Shannon and Simpson indices.

#### Alpha Diversity Related to Repetitive Behavior

We tested the association of alpha diversity measured using Shannon and Simpson indices with RRBI (C,C1, and C2 score), which did not show any significant trend in the previous analysis. A negative correlation with alpha diversity (measured by Shannon and Simpson indices) was found in the C2 score (Shannon: *p* = 0.021 and Simpson: *p* = 0.029; Supplementary Material 3).

## Discussion

We used 16S rRNA high-throughput sequencing technology to obtain the abundance of gut microbial genes with large inter-individual variations and examined the gut microbiota composition correlated with ASD phenotypic features quantitatively for the Chinese population living in southeast-China. Our data suggested that two major subgroups, which were characterized either by the increased abundance of *Veillonella* or *Ruminococcus*, might be correlated with RRBI symptom severity in children with ASD. Cluster 1, with a relatively high abundance of *Veillonella* and a low abundance of *Ruminococcus*, showed more severe RRBI symptoms. However, Cluster 2, with a high abundance of *Ruminococcus* and a low abundance of *Veillonella*, presented milder symptoms of repetitive behaviors.

To our knowledge, this is the first study to show that the composition of the intestinal microbiome is associated with repetitive, stereotyped behaviors in children with ASD. Participants in each group were defined based on the composition of their gut microbiome. Although the use of clustering to analyze the microbiome remains a controversial topic (Knights et al., 2014), this study provides a considerably important translational starting point to reflect the underlying mechanism of autism symptom severity and to explore potential treatment methods.

The genus, *Ruminococcus*, is recognized as the core gut microbiome and is abundant in the human intestinal system. Compared to typically developed children, the abundance of *Ruminococcus torques* is significantly elevated in stool samples from children with autism (Williams et al., 2012; Wang et al., 2013). However, other researchers have reported a decrease in *Ruminococcus* abundance in autism stool samples (Finegold et al., 2010). *Ruminococcus* plays an essential role in boosting intestinal movement and food digestion by degrading mucus. It has been proven that mucin in the large bowel can facilitate the growth of *Ruminococcus torques*, which is also associated with gastrointestinal (GI) disturbance in patients with inflammatory bowel disease (IBD) (Wang et al., 2013). Groups with higher *Ruminococcus* abundance showed less severe repetitive, stereotypical behaviors in our study. The role of *Ruminococcus* spp. in affecting RRBI symptoms and GI symptoms is not clear. *Veillonella* is known to metabolize carbohydrates in the human digestive system and its abundance is decreased in children with ASD in older age groups (Strati et al., 2017). Our study found that toddlers with ASD who carry a higher abundance of *Veillonella* exhibited more severe repetitive, stereotypic behaviors. Thus, further studies are needed to explore the underlying mechanism using metagenome sequencing to describe species in the genera *Ruminococcus* and *Veillonella* quantitatively.

Alpha diversity is an indicator of the richness of species and the diversity of the gut microbiome in a single ecosystem and it reflects local diversity. Lower α-diversity commonly represents a less mature gut microbiome and is often related to unhealthy conditions (Abrahamsson et al., 2014; Kostic et al., 2015). We observed that Cluster 1 had lower alpha diversity and more severe RRBI symptoms (measured by the observed*_*OTUs method*).* In addition, one sub-domain of RRBI, apparently compulsively adhering to non-functional routines or rituals, had a negative correlation with alpha diversity in children with autism (measured by the Shannon and Simpson indices), which is in line with the previously obtained outcomes. Hence, the alteration of alpha-diversity in relation to autism or other developmental delays is still not clear. Some studies have shown that alpha-diversity decreases in patients with ASD (Kang et al., 2013, 2017, 2018), while others show no significant changes (Gondalia et al., 2012; Kushak et al., 2017; Strati et al., 2017), or an increase (Finegold et al., 2010; De Angelis et al., 2013). Further research is needed to target clinical heterogeneity, and a larger population would provide further evidence of the relationship between alpha-diversity and ASD phenotypes.

This is a preliminary study to explore the relationship between composition and alpha-diversity in the gut microbiome and repetitive behavior in ASD. Because of the nature of the unsupervised clustering method, we need to explain the outcomes cautiously and be aware of generalization.

In summary, we demonstrated that the difference in intestinal microbiome composition and altered alpha diversity can be associated with repetitive, stereotypic behavior in autism. The role of *Ruminococcus* and *Veillonella* in ASD is not yet understood.

## Data Availability Statement

The data presented in the study are included in the article/Supplementary Material, further inquiries can be directed to the corresponding authors.

## Ethics Statement

The studies involving human participants were reviewed and approved by the Ethics Committee of Third Hospital of Sun Yat-sen University. Written informed consent to participate in this study was provided by the participants’ legal guardian/next of kin.

## Author Contributions

BC contributed to sample preparation, data collection, and took the lead in writing the manuscript. NY contributed to the interpretation of the results. BP and XH contributed to data analysis. XZ contributed to the leading of all research steps and interpretation of the results. All authors provided critical feedback and helped shape the research, analysis, and manuscript.

## Conflict of Interest

The authors declare that the research was conducted in the absence of any commercial or financial relationships that could be construed as a potential conflict of interest.

## Publisher’s Note

All claims expressed in this article are solely those of the authors and do not necessarily represent those of their affiliated organizations, or those of the publisher, the editors and the reviewers. Any product that may be evaluated in this article, or claim that may be made by its manufacturer, is not guaranteed or endorsed by the publisher.
