# Supplementary material for: Application of Clustering Method to Explore the Correlation Between Dominant Flora and the Autism Spectrum Disorder Clinical Phenotype in Chinese Children
Source: Front Neurosci. 2021 Nov 24;15:760779. doi: 10.3389/fnins.2021.760779 (PMC8652116; doi:10.3389/fnins.2021.760779)
Supplement: Supplementary file 1 [file Image_1.pdf]

Supplement1: Genera difference of Veillonella and Ruminococcus from Cluster 1 and 2 (wunifrac distance)

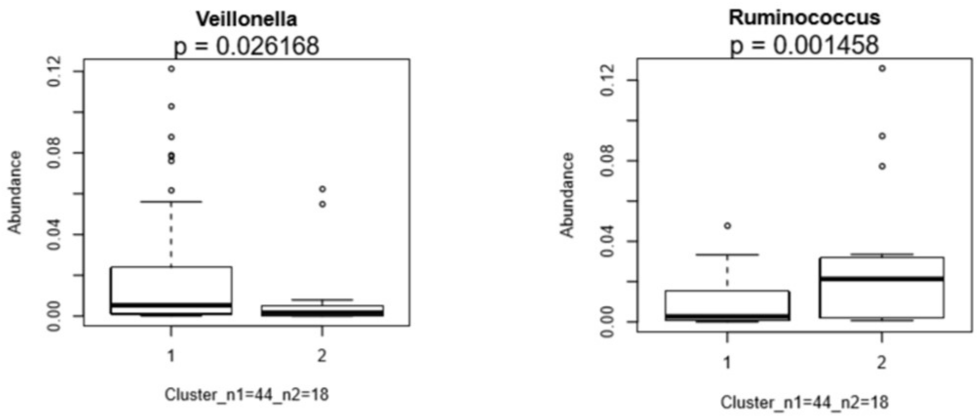

Supplement2-A: ADIR-A score difference between clusters (wunifrac distance)

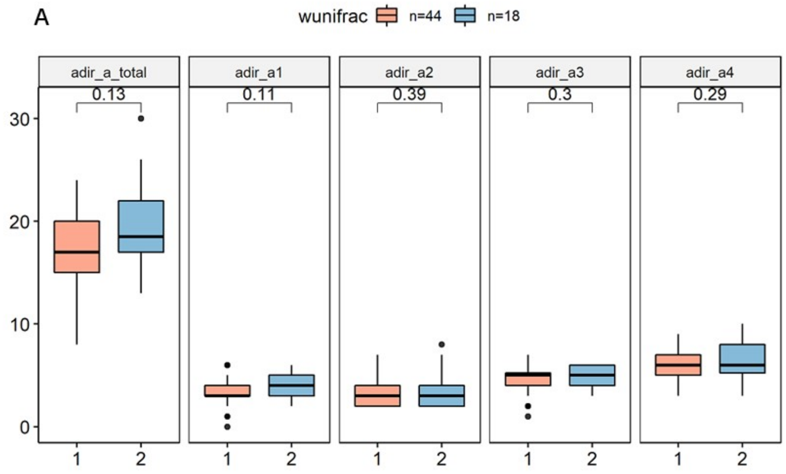

ADI-R: Autism Diagnostic Interview-Revised. ADI-R A score: Social Interaction.
adir_a1: Failure to use nonverbal behaviors to regulate social interaction. adir_a2: Failure to develop peer relationships. adir_a3: Lack of shared enjoyment. adir_a4: Lack of socioemotional reciprocity.

Supplement2-B: ADIR-B score difference between clusters (wunifrac distance)

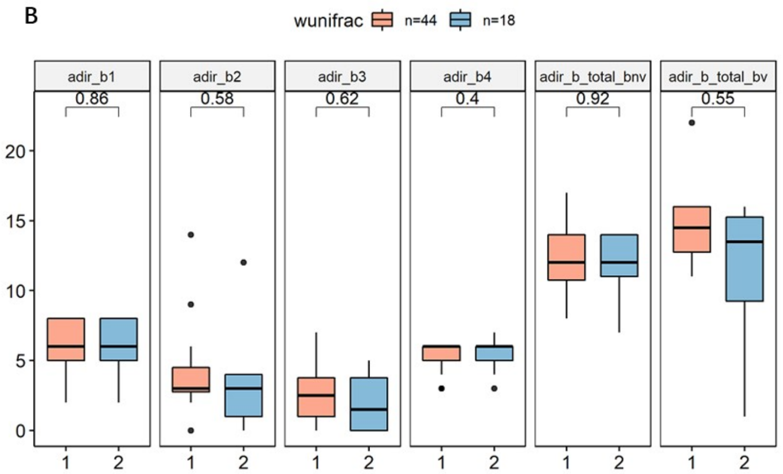

ADI-R: Autism Diagnostic Interview-Revised. ADI-R B score: Communication.
adir_b1: Lack of or delay in spoken language and failure to compensate through gesture. adir_b2: Relative failure to initiate or sustain conversational interchange. adir_b3: Stereotyped, repetitive, or idiosyncratic speech. adir_b4: Lack of varied spontaneous make-believe or social imitative play.

Supplement3: ADIR C2-score correlation with alpha diversity

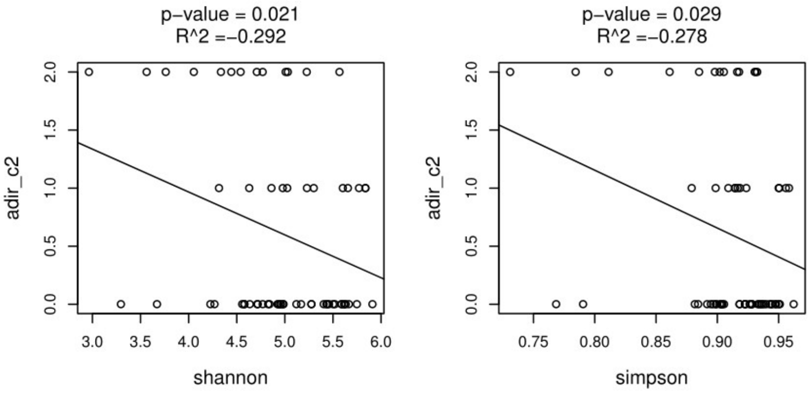

ADI-R: Autism Diagnostic Interview-Revised. ADI-R C2 score: apparently compulsive adherence to nonfunctional routines or rituals.
