# Supplementary material for: Application of Clustering Method to Explore the Correlation Between Dominant Flora and the Autism Spectrum Disorder Clinical Phenotype in Chinese Children
Source: Front Neurosci. 2021 Nov 24;15:760779. doi: 10.3389/fnins.2021.760779 (PMC8652116; doi:10.3389/fnins.2021.760779)
Supplement: Supplementary file 2 [file Table_1.docx]

Supplement 4. Dominant genera in each cluster-Top Ten

| No. | Cluster1 | |  |  | Cluster2 | |  |
| --- | --- | --- | --- | --- | --- | --- | --- |
|  | genus | abundance | p value |  | genus | abundance | p value |
| 1 | Veillonella | 0.020831224 | 0.026167705 |  | Ruminococcus | 0.029954582 | 0.001458475 |
| 2 | Bacteroides | 0.338812244 | 0.073071045 |  | Faecalibacterium | 0.156922744 | 0.003582912 |
| 3 | *Erysipelotrichaceae | 0.005966748 | 0.143879466 |  | Paludibacter | 7.22E-06 | 0.00630136 |
| 4 | [Ruminococcus] | 0.033693883 | 0.152672534 |  | Parvimonas | 2.57E-05 | 0.016183584 |
| 5 | Streptococcus | 0.011522626 | 0.157216643 |  | Pyramidobacter | 0.000398028 | 0.019148057 |
| 6 | Sutterella | 0.020341511 | 0.162611593 |  | *Ruminococcaceae | 0.068386392 | 0.0211966 |
| 7 | Actinomyces | 6.67E-05 | 0.165072605 |  | Adlercreutzia | 2.48E-05 | 0.021409722 |
| 8 | Enterococcus | 0.000291512 | 0.21973901 |  | **RB41 | 1.91E-06 | 0.027538265 |
| 9 | Klebsiella | 0.002534049 | 0.257488345 |  | **MLE1-12 | 1.67E-05 | 0.027538265 |
| 10 | [Eubacterium] | 0.002095896 | 0.351365181 |  | Synechococcus | 1.57778E-06 | 0.027538265 |
|  |  |  |  |  |  |  |  |

‘*’ or ‘**’ indicates the unclassified genera

‘*’ indicates the family to which the genera belongs

‘**’  indicates the order to which the genera belongs

‘[]’ indicates a different genera of genera in ‘[]’

The dominant gut microbiota shown on the table refers to the genera in one cluster with the relative abundance higher than the other group

and p-values of the Wilcoxon signed-rank test between two clusters indicating the significance of differences
